# An Analysis of Organizational Performance Based on Hospital Specialization Level and Strategy Type

**DOI:** 10.1371/journal.pone.0132257

**Published:** 2015-07-28

**Authors:** Han-Sung Kim, Young-Hoon Kim, Jung-Sik Woo, Sook-Jung Hyun

**Affiliations:** 1 Dept. of Healthcare Informatics, Korea Polytechnics, Seoul, Korea; 2 Dept. of Healthcare Management, Graduate School, Eulji University, Gyeonggi, Korea; 3 Dept. of Health Administration, Cheju Halla University, Jeju, Korea; 4 Dept. of Management & Administration, Baekseok Arts University, Seoul, Korea; Azienda Ospedaliero-Universitaria Careggi, ITALY

## Abstract

**Introduction:**

Hospitals are studying the focused factory concept and attempting to increase their power in a competitive industry by becoming more specialized.

**Methodology:**

This study uses the information theory index (ITI) and the Herfindahl-Hirschman index (HHI) to analyze the extent of specialization by Korean hospitals that receive national health insurance reimbursements. Hierarchical regression analysis is used to assess the impact of hospital specialization on the following four aspects of operational performance: productivity, profitability, efficiency and quality of care.

**Study Results:**

The results show that a focused strategy (high HHI) improves the income and adjusted number of patients per specialist through the efficient utilization of human resources. However, a diversified strategy (high ITI) improves the hospital utilization ratio, income per bed and adjusted number of patients per bed (controlling for material resources such as beds). In addition, as the concentration index increases, case-mix mortality rates and referral rates decrease, indicating that specialization has a positive relationship with quality of care.

## Introduction

In the past, hospitals operated in a friendly environment, and the hospital industry was characterized by the provision of physician-oriented services, information asymmetries, limited access to health care and a fee-for-service payment system. In addition, hospitals tended to seek a competitive advantage by expanding the types and number of medical services available because the provision of new medical technologies attracted both physicians and patients [1]. However, hospitals today exist in an environment that is highly uncertain, complex and unpredictable [2]. Accordingly, there has been a continuous emergence of opinions that hospitals should focus on a limited number of services to become more resilient to changes in the health care environment.

For example, Luke, Begun and Walston [3] stated that health care organizations should select and focus on an appropriately limited scope of services. Similarly, Kilduff [4] argued that organizations should focus on a limited number of core businesses and abandon competitively weak business units [4]. In sum, hospitals should focus on specific medical services that meet patients’ needs.

Forty years ago, Skinner [5] proposed the focused factory concept, which argues that businesses should select and focus on a limited number of competitive areas. The focused factory concept has since been suggested as a strategy for the health care industry based on the notion that hospitals can increase the efficiency of patient treatment by focusing on specific diseases or medical procedures [6–10]. For example, Leung [11] argued that the delivery of hospital services through the focused factory model would promote both productivity and efficiency. Moreover, the bankruptcy rate of small and mid-sized hospitals, which collectively play a central role in Korean health care, has increased since the 1997 currency crisis.

To give examples from more recent literature on the effects of focus, Capkun, Messner, and Rissbacher [12] conducted an empirical study and stressed the importance of hospital service specialization in certain types of services away from the integrated-delivery type of health care system, and Lee, Chun, and Lee [13] performed a data envelopment analysis (DEA) and argued for the necessity of reforming the hospital service structure, such as focus-oriented specialization, in order to improve the efficiency of health care.

Thus, this study aims to contribute to hospital competitiveness by conducting a comparative analysis of the degree of specialization in Korean hospitals. The study has two specific purposes: the first is to analyze the type and degree of hospital specialization using a specialization measurement tool, and the second is to analyze the influence of the degree of specialization on four aspects of organizational performance: profitability, efficiency, productivity and quality of care.

## Methodology

### Selection of hospitals for analysis

This study gathered data for 1,810 target hospitals that had more than 30 beds and that claimed health insurance reimbursement in 2011 using merged data from Statistics Korea and the Korean Hospital Association. After excluding 375 hospitals that opened or closed during 2011 (353 hospitals) and that had less than 100 inpatient insurance claims during that period (20 hospitals), 1,437 target hospitals. In terms of hospital type, hospital accounted for the majority of the sample, with 1,131 facilities. There were 825 privately owned hospitals in the sample, more than any other ownership type. Hospitals with fewer than 300 beds accounted for 1,207 hospitals in the sample, and hospitals with fewer than 10 specialists accounted for 970 hospitals. More hospitals were located in cities (426) than in any other type of location, and 698 hospitals had been established for fewer than 5 years. There were more non-teaching hospitals than teaching hospitals and more full-service hospitals than specialty hospitals (cardiovascular surgery e.g.). Additionally, most hospitals in the sample did not participate in the diagnosis-related group (DRG) payment scheme.

### Measure of specialization

This study measured hospital specialization by calculating the Herfindahl-Hirschman index (HHI) of Zwanziger, Melnick and Simonson [14] and the information theory index (ITI) of Evans and Walker [15] based on Korean diagnosis-related groups (KDRGs). ITI is a relative concept that reflects the level of diversification in the service mix provided by a particular hospital by comparing it to the average service mix provided by all hospitals in the sample, whereas HHI is an absolute concept that represents the concentration of the service mix provided by a particular hospital.

The closer the ITI score to zero, the higher the similarity between the service mixes provided by all hospitals and a particular hospital, and the higher the ITI score of a particular hospital, the higher the diversification of that hospital. In contrast, the closer the HHI score (range: 0–1) to 1, the higher the degree of focusing on specialized services.

### Control variables

Hospital specialization and organizational performance are influenced by various environmental factors [3]. Previous studies have considered the impact of market competition [16–18], population density [17], the number of doctors in the region [16,19], competitor characteristics [20] and income [16–18]. Internal organizational characteristics are also important determinants of an organization’s goals and performance and likewise affect organizational behavior, including the implementation of specialization strategies [21]. Internal organizational characteristics such as size, location, ownership, control, system membership, goals, age and life-cycle stage are regarded as important factors in strategic decision making [22]. In previous health care studies, organizational characteristics such as the number of beds [23], patient type [24], ownership [16], market share [18] and payment system [17] have been considered. The environmental and organizational characteristics used as control variables in this study are the same as those used in previous studies (Table 1).

**Table 1:** Measures and Data Sources Used to Operationalize Variables.

Factors	Variables	Measurement	Attribution	Data Sources
External Environment	Regional Competitors	Number of regional hospitals	Continuous	KOSTAT: Regional Statistics Data
	Regional Physicians	(Physicians ÷ Population) × 1,000	Continuous	KOSTAT: Regional Statistics Data
	Regional Population	Regional population	Continuous	KOSTAT: Regional Statistics Data
	Aged Population Ratio (%)	Population aged 65↑ ÷ regional population	Continuous	KOSTAT: Regional Statistics Data
	Regional Income per Capita	Gross regional domestic product ÷ regional population	Continuous	KOSTAT: Regional Statistics Data
Internal Environment	Hospital Size	Number of beds	Continuous	KHA/ HIRA: Hospital Resource Data
	Hospital Type	Hospital type (hospital = 0, general = 1, tertiary = 2)	Categorical	KHA/ HIRA: Hospital Resource Data
	Hospital Age	Age of hospital (2011 establishment year)	Continuous	KHA/ HIRA: Hospital Resource Data
	Ownership	Hospital ownership (public = 0, university = 1, other = 2, private = 3)	Categorical	KHA/ HIRA: Hospital Resource Data
	Hospital Location	Hospital location type (rural = 0, mid-sized = 1, urban = 2, megalopolis = 3, metropolitan = 4)	Categorical	KHA/ HIRA: Hospital Resource Data
	Teaching Hospitals	Intern and resident education Y/N (N = 0, Y = 1)	Categorical	KHA/ HIRA: Hospital Resource Data
	Specialty Hospitals	Specialty assigned by hospital Y/N (N = 0, Y = 1)	Categorical	KHA/ HIRA: Hospital Resource Data
	Number of Physicians	Number of specialists	Continuous	KHA/ HIRA: Hospital Resource Data
	DRG Participation	7-DRG participation Y/N (N = 0, Y = 1)	Continuous	KHA/ HIRA: Hospital Resource Data
Specialization Index	Diversification	ITI score	Continuous	HIRA: Inpatient Insurance Claim (DRG Tagging)
	Concentration	HHI score	Continuous	
Organizational Performance	Income per Specialist	Total income ÷ number of specialists	Continuous	HIRA: National Health Claims Data
	Adjusted Income per Patient	Total income ÷ adjusted number of patients	Continuous	HIRA: National Health Claims Data
	Income per Bed	Total income ÷ number of beds	Continuous	HIRA: National Health Claims Data
	Adjusted Number of Patients per Specialist	Adjusted number of patients ÷ number of specialists	Continuous	HIRA: National Health Claims Data
	Adjusted Number of Patients per Bed	Adjusted number of patients ÷ number of beds	Continuous	HIRA: National Health Claims Data
	Length of Stay	Days at hospital ÷ number of inpatients	Continuous	HIRA: National Health Claims Data
	Hospital Utilization (%)	Adjusted number of patients ÷ (beds × 365)	Continuous	HIRA: National Health Claims Data
	Bed Utilization (%)	Number of hospitalized patients ÷ (beds × 365)	Continuous	HIRA: National Health Claims Data
	Adjusted Referral Index	(Adjusted DRG) actual referral rate ÷ expected referral rate	Continuous	HIRA: National Health Claims Data
	Adjusted Mortality Index	(Adjusted DRG) actual mortality ÷ expected mortality	Continuous	HIRA: National Health Claims Data
	Severity Index	Complexity disease group ratio—simple disease group ratio	Continuous	HIRA: National Health Claims Data

### Measure of performance

Various indicators have been used to measure hospital performance in previous studies, including income, cost per patient, cost per inpatient, operating margin, return on assets (ROA) and return on investment (ROI) [18,23,25–27]. However, these performance measures may be inappropriate for evaluating the success (or failure) of a change in strategy [28]. Fahey and Christensen [29] argued that it is particularly important to know what a strategy was intended to achieve when evaluating its effectiveness. For example, specialization is intended not only to achieve a competitive advantage but also to reduce costs. To reduce costs, productivity must be improved, and input costs must be decreased. Moreover, a competitive advantage depends not only on reduced costs and increased profits but also on enhanced quality of care. A specialization strategy that improves efficiency and productivity may also enhance the quality of care provided. Thus, hospitals that achieve improvements in productivity, efficiency and quality of care gain a competitive advantage over other hospitals. This study uses four performance indicators to represent the effects of specialization: profitability, efficiency, productivity and quality of care. (Table 1)

The limitation of this study in this regard is that no analysis based on financial information could be conducted for want of cost report data among the variables of organizational performance.

### Method of analysis

**Fig 1:**
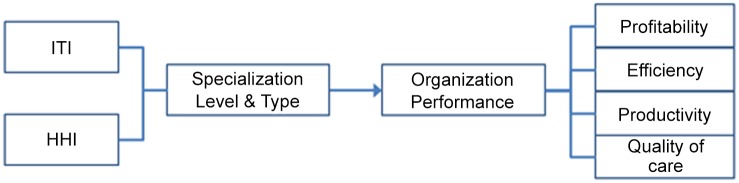
Research Framework.

The extent of hospital specialization was measured by calculating the concentration and level of diversification of each hospital’s service mix, and the types of hospital specialization strategies were then classified using cluster analysis. However, before conducting the analysis of hospital specialization, correlation analyses were performed to test for multicollinearity among the variables representing the internal and external environments. Multicollinearity between continuous variables was diagnosed using the Pearson correlation coefficient (Pearson’s r); multicollinearity between continuous and categorical variables was diagnosed using the point biserial correlation coefficient; and multicollinearity between categorical variables was diagnosed using phi and Cramer’s V coefficients. Tabachnick and Fidell [30] found that a multicollinearity problem exists when the absolute value of correlation coefficient r is higher than 0.7; they argued that in such cases, one of the two variables should be excluded (Fig 1).

After variables exhibiting high levels of multicollinearity with each other were excluded, hierarchical regression analysis was performed to investigate the relationship between specialization strategy and organizational performance. During the first phase of the hierarchical regression analysis, the control variables representing environmental and organizational characteristics were input. During the second phase, the variable representing the type of specialization strategy adopted by each hospital was input. The analytic tool SAS 9.1 was used to process the data (e.g., to calculate the specialization and performance indices), and SPSS 18.0 was used to calculate the descriptive statistics and to perform cross tabulation, cluster analysis, two-way ANOVA and hierarchical regression analysis.

As such, this study adopted a cross-sectional study design; in the future, however, an elaborate analysis of the degree of shift to specialization and the change in organizational performance will have to be pursued on the basis of a longitudinal study design.

## Study Results

### The effect of specialization on organizational performance

The results relating to productivity indicate that the adjusted number of patients per specialist is highly positively correlated with the concentration index. In other words, hospitals with high HHIs exhibited high productivity per medical service unit. Moreover, although there was no statistically significant relationship between the diversification index and the adjusted number of patients per specialist, there was a positive relationship between the diversification index and the adjusted number of patients per bed. Therefore, increases in the HHIs and ITIs of medical services had a positive effect on productivity (Table 2).

**Table 2:** Hierarchical Regression Analysis of the Effect of the Specialization Index on Productivity.

Independent Variable		Adjusted Number of Patients per Specialist		Adjusted Number of Patients per Bed	
		[Model 1] Sd. Coef.	[Model 2] Sd. Coef.	[Model 1] Sd. Coef.	[Model 2] Sd. Coef.
Regional Competitors		0.000	-0.006	0.040	0.036
Regional Physicians		-0.021	-0.019	-0.050	-0.057 *
Aged Population Ratio (%)		0.119 **	0.131 **	0.025	0.024
Regional Income per Capita		-0.023	-0.014	0.023	0.024
Hospital Size		0.371 **	0.369 **	-0.117 **	-0.148 **
Hospital Age		0.021	0.004	0.010	0.023
Hospital Type^1^ ^)^	General	-0.319 **	-0.216 **	0.202 **	0.199 **
	Tertiary	-0.303 **	-0.227 **	0.258 **	0.242 **
Ownership^2^ ^)^	University	-0.057	-0.066	-0.032	-0.022
	Other	0.015	0.007	0.034	0.043
	Private	-0.041	-0.031	0.093	0.099
Hospital Resident^3^ ^)^	Mid-sized	0.096	0.067	-0.034	-0.027
	Urban	0.047	0.019	-0.026	-0.028
	Megapolis	0.096	0.064	-0.022	-0.017
	Metropolitan	-0.049	-0.065	-0.071	-0.075
DRG Participation^4^ ^)^		-0.150 **	-0.154 **	0.125 **	0.110 **
Teaching Hospitals^5^ ^)^		-0.119 **	-0.101 **	0.183 **	0.167 **
Specialty Hospitals^5^ ^)^		-0.085 **	-0.092 **	0.068 **	0.057 *
ITI			-0.044		0.064 *
HHI			0.271 **		-0.029
ITI×HHI			-0.076 **		0.056
Statistics		R^2^ = 0.250	R^2^ = 0.311 (Δ0.061**)	R^2^ = 0.135	R^2^ = 0.143 (Δ0.008**)
		F = 26.246	F = 30.433	F = 12.324	F = 11.282
		p = 0.000	p = 0.000	p = 0.000	p = 0.000

Note

^1)^ Hospital Type: Hospital (reference),

^2)^ Ownership: Public (reference),

^3)^ Hospital Resident: Rural (reference),

^4)^ DRG: Non-participation (reference),

^5)^ Teaching/Specialty Hospitals: Non-Assigned (reference) 6)

*p<0.05,

**p<0.01

With respect to profitability, income per specialist increased as the concentration index increased. Thus, income per specialist was high at hospitals with high HHIs. There was no statistically significant relationship between the diversification index and adjusted income per patient; however, similar to the results relating to productivity, there was a positive relationship between the diversification index and income per bed. In addition, the concentration index had a negative effect on income per bed (similar to the negative effect of concentration on the adjusted number of patients per bed). In sum, service-mix diversification had a positive effect on productivity and profitability per bed, whereas service-mix concentration had a positive effect on productivity and profitability per specialist (Table 3).

**Table 3:** Hierarchical Regression Analysis of the Effect of the Specialization Index on Profitability.

Independent Variable		Income per Specialist		Adjusted Income per Patient		Income per Bed	
		[Model 1] Sd. Coef.	[Model 2] Sd. Coef.	[Model 1] Sd. Coef.	[Model 2] Sd. Coef.	[Model 1] Sd. Coef.	[Model 2] Sd. Coef.
Regional Competitors		-0.035	-0.040	0.050 *	0.034	0.048 *	0.038
Regional Physicians		0.009	0.006	0.060 *	0.039	0.020	0.003
Aged Population Ratio (%)		0.106 *	0.109 *	-0.083 *	-0.087 **	-0.062 *	-0.067 *
Regional Income per Capita		-0.010	-0.006	0.002	0.006	0.005	0.005
Hospital Size		0.308 **	0.309 **	-0.056	-0.087 **	0.031	-0.018
Hospital Age		-0.026	-0.028	-0.102 **	-0.077 **	-0.075 **	-0.046 *
Hospital Type^1^ ^)^	General	-0.161 **	-0.119 **	0.137 **	0.170 **	0.213 **	0.202 **
	Tertiary	-0.143 **	-0.112 **	0.283 **	0.294 **	0.407 **	0.378 **
Ownership^2^ ^)^	University	-0.030	-0.033	0.158 **	0.170 **	0.111 **	0.130 **
	Other	0.104	0.099	0.229 **	0.231 **	0.176 **	0.189 **
	Private	0.138 *	0.141 *	0.263 **	0.270 **	0.233 **	0.242 **
Hospital Resident^3^ ^)^	Mid-sized	0.137 *	0.127 *	-0.053	-0.048	-0.070	-0.055
	Urban	0.157 **	0.144 *	0.039	0.020	0.016	0.012
	Megapolis	0.174 **	0.161 *	-0.005	-0.012	-0.042	-0.034
	Metropolitan	0.007	-0.003	0.124 **	0.099 *	0.055	0.043
DRG Participation^4^ ^)^		-0.131 **	-0.142 **	0.241 **	0.184 **	0.201 **	0.161 **
Teaching Hospitals^5^ ^)^		0.012	0.008	0.293 **	0.236 **	0.307 **	0.261 **
Specialty Hospitals^5^ ^)^		0.011	-0.006	0.210 **	0.147 **	0.193 **	0.156 **
ITI			-0.040		-0.018		0.077 **
HHI			0.086 **		-0.027		-0.099 **
ITI×HHI			0.041		0.304 **		0.205 **
Statistics		R^2^ = 0.116	R^2^ = 0.125 (Δ0.009**)	R^2^ = 0.433	R^2^ = 0.504 (Δ0.072**)	R^2^ = 0.567	R^2^ = 0.618 (Δ0.051**)
		F = 10.335	F = 9.644	F = 60.094	F = 68.532	F = 103.188	F = 108.875
		p = 0.000	p = 0.000	p = 0.000	p = 0.000	p = 0.000	p = 0.000

Note

^1)^ Hospital Type: Hospital (reference),

^2)^ Ownership: Public (reference),

^3)^ Hospital Resident: Rural (reference),

^4)^ DRG: Non-participation (reference),

^5)^ Teaching/Specialty Hospitals: Non-Assigned (reference) 6)

*p<0.05,

**p<0.01

The results described above suggest that diversification may be classified as a strategy that improves productivity and profitability by maximizing the effective use of beds (divided broadly into medical facility), whereas concentration may be classified as a strategy that improves productivity and profitability by maximizing the effective use of physicians, who are the primary providers of medical services.

The results relating to the efficiency aspect of organizational performance indicated that average length of stay sharply increased when the concentration index increased. Because length of stay has long been viewed as a negative influence on efficient bed utilization, the concentration index was found to have a negative effect on the bed utilization rate. There was no statistically significant relationship between the diversification index and either average length of stay or (inpatient) bed utilization ratio; however, a positive relationship was found to exist between the diversification index and hospital utilization rates that included outpatients. In other words, the higher the diversification index was, the higher the hospital utilization rate was (Table 4).

**Table 4:** Hierarchical Regression Analysis of the Effect of the Specialization Index on Efficiency.

Independent Variable		Length of Stay		Hospital Utilization		Bed Utilization	
		[Model 1] Sd. Coef.	[Model 2] Sd. Coef.	[Model 1] Sd. Coef.	[Model 2] Sd. Coef.	[Model 1] Sd. Coef.	[Model 2] Sd. Coef.
Regional Competitors		-0.025	-0.029	0.058	0.051	0.040	0.036
Regional Physicians		-0.013	-0.005	-0.059 *	-0.065 *	-0.050	-0.057 *
Aged Population Ratio (%)		0.046	0.063	0.109 *	0.113 *	0.025	0.024
Regional Income per Capita		-0.059 *	-0.049 *	-0.029	-0.024	0.023	0.024
Hospital Size		0.432 **	0.426 **	0.177 **	0.160 **	-0.117 **	-0.148 **
Hospital Age		0.078 **	0.051 *	-0.015	-0.013	0.010	0.023
Hospital Type^1^ ^)^	General	-0.264 **	-0.134 **	0.139 **	0.194 **	0.202 **	0.199 **
	Tertiary	-0.345 **	-0.251 **	0.131 **	0.164 **	0.258 **	0.242 **
Ownership^2^ ^)^	University	-0.139 **	-0.151 **	-0.039	-0.037	-0.032	-0.022
	Other	-0.234 **	-0.242 **	0.051	0.050	0.034	0.043
	Private	-0.381 **	-0.367 **	0.086	0.094	0.093	0.099
Hospital Resident^3^ ^)^	Mid-sized	0.120 *	0.081	0.112 *	0.101	-0.034	-0.027
	Urban	0.056	0.023	0.117 *	0.100	-0.026	-0.028
	Megapolis	0.093	0.053	0.141 *	0.126	-0.022	-0.017
	Metropolitan	0.007	-0.007	0.046	0.031	-0.071	-0.075
DRG Participation^4^ ^)^		-0.269 **	-0.258 **	0.058 *	0.039	0.125 **	0.110 **
Teaching Hospitals^5^ ^)^		-0.198 **	-0.158 **	0.087 *	0.078 *	0.183 **	0.167 **
Specialty Hospitals^5^ ^)^		-0.056 *	-0.046 *	0.114 **	0.093 **	0.068 **	0.057 *
ITI			-0.020		-0.004		0.064 *
HHI			0.379 **		0.114 **		-0.029
ITI×HHI			-0.204 **		0.045		0.056
Statistics		R^2^ = 0.309	R^2^ = 0.435 (Δ0.126**)	R^2^ = 0.132	R^2^ = 0.146 (Δ0.014**)	R^2^ = 0.135	R^2^ = 0.143 (Δ0.008**)
		F = 35.272	F = 51.917	F = 11.943	F = 11.508	F = 12.324	F = 11.282
		p = 0.000	p = 0.000	p = 0.000	p = 0.000	p = 0.000	p = 0.000

Note

^1)^ Hospital Type: Hospital (reference),

^2)^ Ownership: Public (reference),

^3)^ Hospital Resident: Rural (reference),

^4)^ DRG: Non-participation (reference),

^5)^ Teaching/Specialty Hospitals: Non-Assigned (reference) 6)

*p<0.05,

**p<0.01

The results relating to quality of care showed that the severity index decreased as the concentration index increased, which indicates that hospitals with high HHIs provide medical services to patients of low severity. DRG-adjusted mortality and referral rates had a highly positive relationship with ITI levels that indicated increased specialization; therefore, mortality and referral rates decreased as the concentration index increased. In sum, a higher level of specialization had a positive effect on referral and mortality rates (Table 5).

**Table 5:** Hierarchical Regression Analysis of the Effect of the Specialization Index on Quality of Care.

Independent Variable		Adjusted Referral Index		Adjusted Mortality Index		Severity Index	
		[Model 1] Sd. Coef.	[Model 2] Sd. Coef.	[Model 1] Sd. Coef.	[Model 2] Sd. Coef.	[Model 1] Sd. Coef.	[Model 2] Sd. Coef.
Regional Competitors		0.012	0.011	-0.045	-0.039	0.004	0.011
Regional Physicians		0.076**	0.073 **	-0.066 *	-0.063 *	0.008	0.011
Aged Population Ratio (%)		-0.058	-0.061	0.046	0.039	0.024	0.015
Regional Income per capita		-0.040	-0.041	-0.019	-0.025	0.027	0.020
Hospital Size		0.349 **	0.360 **	-0.025	-0.011	-0.012	0.023
Hospital Age		-0.014	-0.010	0.114 **	0.119 **	0.080 *	0.081 *
Hospital Type^1^ ^)^	General	0.069 *	0.053	0.245 **	0.172 **	0.153 **	0.079 *
	Tertiary	0.047	0.040	0.123 **	0.075	0.042	0.001
Ownership^2^ ^)^	University	0.053	0.052	-0.017	-0.015	-0.055	-0.058
	Other	-0.017	-0.020	-0.020	-0.018	-0.026	-0.030
	Private	-0.046	-0.050	-0.105	-0.114	-0.033	-0.047
Hospital Resident^3^ ^)^	Mid-sized	-0.063	-0.057	-0.119 *	-0.101	-0.114	-0.098
	Urban	-0.040	-0.037	-0.157 **	-0.135 *	-0.210 **	-0.187 **
	Megapolis	-0.048	-0.044	-0.148 *	-0.127	-0.192 **	-0.172 *
	Metropolitan	-0.040	-0.041	-0.097	-0.083	-0.190 **	-0.175 **
DRG Participation^4^ ^)^		-0.068 **	-0.076 **	0.002	0.014	0.035	0.048
Teaching Hospitals^5^ ^)^		0.070 *	0.057	-0.100 **	-0.102 **	-0.096 *	-0.098 *
Specialty Hospitals^5^ ^)^		0.027	0.016	-0.006	0.008	-0.018	-0.008
ITI			-0.036		0.010		-0.047
HHI			-0.067 *		-0.175 **		-0.190 **
ITI×HHI			0.084 **		0.008		0.039
Statistics		R^2^ = 0.266	R^2^ = 0.272 (Δ0.006**)	R^2^ = 0.131	R^2^ = 0.155 (Δ0.025**)	R^2^ = 0.067	R^2^ = 0.095 (Δ0.028**)
		F = 28.528	F = 25.182	F = 11.832	F = 12.385	F = 5.666	F = 7.043
		p = 0.000	p = 0.000	p = 0.000	p = 0.000	p = 0.000	p = 0.000

Note

^1)^ Hospital Type: Hospital (reference),

^2)^ Ownership: Public (reference),

^3)^ Hospital Resident: Rural (reference),

^4)^ DRG: Non-participation (reference),

^5)^ Teaching/Specialty Hospitals: Non-Assigned (reference) 6)

*p<0.05,

**p<0.01

### Comparison of the effects of specialization strategy types on organizational performance

K-means cluster analysis was performed using ITI and HHI—the two specialization indices that were calculated based on the 1,437 sample hospitals—as the cluster variables. Because the ITI and HHI values did not exhibit a normal distribution, they were log transformed before being input into the cluster analysis (Table 6).

**Table 6:** Cluster Analysis of Specialization Strategy Type.

Specialization Index	Cluster Type				F-value	p
	Cluster (1)	Cluster (2)	Cluster (3)	Cluster (4)		
ITI	0.313	0.092	0.666	7.280	1234.2	0.000
HHI	0.040	0.275	0.217	0.019	1463.5	0.000
Number of Hospitals	595	424	275	143		
Classification	General Type	Concentrated Type	Integrated Type	Diversified Type		

Note: ITI, HHI index average is not log transformed.

The results of the cluster analysis showed that R-squared values greatly increased up to four cluster groups, after which point R-squared growth decreased. Thus, the optimal number of cluster groups was four, with an R-squared value of 75.4%.

As a result of K-means cluster analysis, both the diversification index and the concentration index—which were the two specialization indices among clusters—demonstrated statistically significant differences. The first cluster comprised 595 hospitals that did not display diversification or concentration superiority; this cluster was classified as having a general strategy. The second cluster, comprising 424 hospitals, had the lowest diversification index and the highest concentration index. Therefore, the second cluster was classified as a group that pursued a concentrated service strategy. The third cluster included 275 hospitals with mid-level HHIs and ITIs; thus, it was classified as a group that pursued an integrated strategy. The fourth cluster, comprising 143 hospitals, had the highest diversification index and the lowest concentration index. Thus, the fourth cluster was classified as a group that pursued a diversified service strategy. These four specialization strategy clusters are not absolute clusters; rather, they are relative clusters based on the two specialization indices. Thus, the general strategy type of the first cluster represents a strategy that is relatively low in terms of the two specialization indices.

ANOVA was conducted to evaluate the relationship between strategy type and organizational performance. With respect to quality of care, the results showed that, relative to the general strategy cluster, the diversified strategy cluster had a high severity index and the concentrated and integrated strategy clusters had low referral and mortality rates. With respect to efficiency, the diversified strategy cluster had a short length of stay and high bed and hospital utilization rates relative to the other strategy types. Productivity per specialist was found to be high in the concentrated strategy cluster, whereas productivity per bed was found to be high in the diversified strategy cluster. With respect to profitability, Scheffe’s post-hoc test indicated that there was no difference among strategy types in terms of income per specialist; however, adjusted income per patient and profit per bed were found to be higher in the diversified strategy cluster (Table 7).

**Table 7:** Differences in Organizational Performance among Specialization Strategy Types.

Strategic Type Performance		General		Concentrated		Integrated		Diversified		F-value
		Mean	(S.D.)	Mean	(S.D.)	Mean	(S.D.)	Mean	(S.D.)	
Productivity	Adjusted number of patients per specialist	7356	(4745)^a^	12923	(8953)^b^	8506	(11447)^a^	3553	(1829)^c^	70.855**
	Adjusted number of patients per bed	389	(132)^a^	356	(192)^a^	452	(214)^b^	562	(131)^c^	61.651**
Profitability	Income per specialist^3^ ^)^	72.2	(32.4)^b^	79.5	(44.6)^a^	79.3	(51.0)^a^	75.8	(18.9)^b^	3.620*
	Adjusted income per patient^4^ ^)^	109.8	(36.6)^a^	80.0	(62.2)^b^	154.4	(117.3)^c^	240.0	(82.6)^d^	207.449**
	Income per bed^5^ ^)^	4.3	(2.1)^a^	2.7	(2.1)^a^	6.7	(4.6)^c^	13.6	(5.8)^d^	434.139**
Efficiency	Length of stay	10.8	(3.1)^a^	15.8	(7.8)^a^	9.7	(7.0)^a^ ^,^ ^c^	8.6	(2.2)^c^	107.274**
	Bed utilization (%)	67.0	(24.0)^a^	66.6	(28.8)^a^	86.4	(39.6)^b^	98.2	(16.5)^c^	73.545**
	Hospital utilization (%)	106.6	(36.2)^a^	97.6	(52.5)^a^	123.9	(58.7)^b^	154.0	(35.9)^c^	61.651**
Quality of Care	Severity index	-38.0	(12.1)^a^	-37.8	(32.5)^a^	-32.6	(25.5)^a^	-1.5	(20.2)^b^	103.227**
	Adjusted mortality index	0.9	(0.8)^a^	0.3	(0.8)^b^	0.3	(1.3)^b^	1.0	(0.4)^a^	53.062**
	Adjusted referral index	1.3	(1.7)^a^	0.5	(1.4)^b^	0.7	(1.2)^b^	0.7	(1.0)^b^	31.344**
Strategic Type Performance		General		Concentrated		Integrated		Diversified		F-value
		Mean	(S.D.)	Mean	(S.D.)	Mean	(S.D.)	Mean	(S.D.)	
Productivity	Adjusted number of patients per specialist	7356	(4745)^a^	12923	(8953)^b^	8506	(11447)^a^	3553	(1829)^c^	70.855**
	Adjusted number of patients per bed	389	(132)^a^	356	(192)^a^	452	(214)^b^	562	(131)^c^	61.651**
Profitability	Income per specialist^3^ ^)^	72.2	(32.4)^b^	79.5	(44.6)^a^	79.3	(51.0)^a^	75.8	(18.9)^b^	3.620*
	Adjusted income per patient^4^ ^)^	109.8	(36.6)^a^	80.0	(62.2)^b^	154.4	(117.3)^c^	240.0	(82.6)^d^	207.449**
	Income per bed^5^ ^)^	4.3	(2.1)^a^	2.7	(2.1)^a^	6.7	(4.6)^c^	13.6	(5.8)^d^	434.139**
Efficiency	Length of stay	10.8	(3.1)^a^	15.8	(7.8)^a^	9.7	(7.0)^a^ ^,^ ^c^	8.6	(2.2)^c^	107.274**
	Bed utilization (%)	67.0	(24.0)^a^	66.6	(28.8)^a^	86.4	(39.6)^b^	98.2	(16.5)^c^	73.545**
	Hospital utilization (%)	106.6	(36.2)^a^	97.6	(52.5)^a^	123.9	(58.7)^b^	154.0	(35.9)^c^	61.651**
Quality of Care	Severity index	-38.0	(12.1)^a^	-37.8	(32.5)^a^	-32.6	(25.5)^a^	-1.5	(20.2)^b^	103.227**
	Adjusted mortality index	0.9	(0.8)^a^	0.3	(0.8)^b^	0.3	(1.3)^b^	1.0	(0.4)^a^	53.062**
	Adjusted referral index	1.3	(1.7)^a^	0.5	(1.4)^b^	0.7	(1.2)^b^	0.7	(1.0)^b^	31.344**

Note 1)

**p<0.01

*p<0.05,

^2)^ Post-Hoc Method: Scheffe (subgroup: a, b, c, d ※ treatment income per specialist: Duncan)

^3)^ Korean $ 10,000,000 (₩)

^4)^ Korean $ 1,000 (₩)

^5)^ Korean $ 10,000,000 (₩)

## Conclusions and Implications

Hospital organizations have adopted a new production system, one that is very different from the system used in the past, to survive in the face of environmental changes.

The method typically used to implement these changes was the focused factory approach, which aims for competitive differentiation based on a concentrated range of products rather than the diversification of business units [5,31–33].

In addition, the focused factory concept allows hospitals to meet patient demand for new and specialized services. Specifically, in response to patient demand, hospitals have started to establish patient-oriented, independent specialty centers, as well as in-hospital specialty departments [34–40]. Furthermore, a structural reform of hospital organization is necessary in order to enhance the achievements resulting from specialization [15]. The specialization-mediated improvement of organizational performance should hence be preceded by an organization design tailored to specialization. In this context, Vera and Kuntz [see the reference in the Introduction section above] conducted an empirical study and argued for the implementation of process-based organization design to enhance the efficiency of hospital operation.

However, there are two opposing views on hospital specialization. One side argues that hospital specialization ensures high quality of care and improves efficiency by increasing productivity [41–43]. The other side argues that the survival of general hospitals is threatened by specialized hospitals because specialized hospitals tend to focus on high-margin services and avoid the high expenses related to admitting intensive care patients [44–49].

Therefore, this study calculated two specialization indices (diversification and concentration) to measure the extent of medical service specialization at 1,437 medical institutions at the hospital level or higher. Additionally, this study investigated the relationship between organizational performance and specialization level and type.

The results of this analysis indicate that specialization has a generally positive effect on four aspects of hospital performance. The impact of specialization on organizational performance is described more specifically below.

First, with respect to productivity, the HHI has a positive effect on the adjusted number of patients per specialist, whereas the ITI has a positive effect on the adjusted number of patients per bed. In other words, a high HHI is indicative of high workforce productivity, whereas a high ITI is indicative of high productivity per facility unit (e.g., beds) and capital investment.

Second, with respect to profitability, HHI was found to have a significant positive effect on income per specialist; however, HHI was found to have a negative effect on income per bed. By contrast, ITI had a positive effect on income per bed. In other words, diversification improves productivity and profitability by maximizing the effective use of beds, whereas concentration improves productivity and profitability by maximizing the effective use of physicians, who are the main providers of medical services. The adjusted income per patient was not related to either ITI or HHI; however, the interaction of the two specialization indices was found to have a positive effect on profitability.

Third, with respect to efficiency, although increases in HHI had a negative effect on efficiency, the interaction of ITI and HHI was found to decrease the length of hospital stays. Higher HHIs may be associated with longer hospital stays due to the use of medical resources by hospitals with high HHIs. For example, at relatively small hospitals, there may be an excess of unused medical equipment and beds, which facilitates longer hospital stays. In addition, psychiatric hospitals are included in the cluster of hospitals with high HHIs. The analysis also showed that HHI had a positive effect on (inpatient) bed utilization rates, whereas ITI had a positive effect on bed utilization rates that included both inpatients and outpatients.

Fourth, with respect to quality of care, HHI was found to have a negative relationship with the severity index, which suggests that hospitals with high HHIs provide medical services to patients of low severity. However, the specialization index that combines HHI and ITI appears to have a positive relationship with the proportion of patients of high severity. HHI was found to have a negative relationship with DRG-adjusted mortality and referral rates; in other words, mortality and referral rates decreased as HHI increased. Thus, the results of this study indicate that medical service specialization improves mortality and referral rates.

This study has several implications.

First, this study may help hospital managers to understand the relationships among specialization strategy, environmental changes and performance. The pursuit of a hospital specialization strategy requires the selection of the appropriate type (differentiation) and range (concentration) of services [3,50,51]. Daft [52] argued that it is important for managers to define organizational goals and to evaluate the internal and external environments of the organization before implementing a new business strategy. Therefore, this study is expected to provide information to CEOs and managers of hospital organizations about the effects, directions and contents of the various specialization strategy types.

Second, this study may also provide important guidance to health policy makers who seek to improve the efficiency of hospital systems in the face of increasing national health expenditures. Eastaugh [53] argued that the rational behavior of specialty hospitals is closely related to the payment system used. In particular, he argued that there is no motive to decrease costs under the fee-for-service system, whereas prospective payment systems (such as DRG-based systems) encourage cost efficiency. In Korea, the prospective payment system for seven DRGs was implemented at clinics and hospitals in July 2012 and at general and tertiary hospitals in July 2013. The DRG scheme is scheduled to expand further in the future. Policy makers must implement the DRG-based prospective payment system at specialty hospitals in order to maximize the reduction of medical expenses and achieve cost-efficiency in the health care industry. Moreover, specialty hospitals may find it relatively easy to implement the DRG payment system because the variation in DRGs at these facilities should be relatively low.

Accordingly, this study may provide useful information regarding the measurement and evaluation of specialty hospital performance.
